# Secondary Ophthalmic Features Represent Diagnostic Clues and Potential Points of Intervention for Inherited Retinal Diseases (Target 5000 Report 3)

**DOI:** 10.3390/genes16121433

**Published:** 2025-12-01

**Authors:** Kirk A. J. Stephenson, Julia Zhu, Marcus Conway, Bridget Moran, Adrian Dockery, Laura Whelan, Jacqueline Turner, James J. O’Byrne, D. Ian Flitcroft, G. Jane Farrar, David J. Keegan

**Affiliations:** 1 Mater Clinical Ophthalmic Genetics Unit, Mater Misericordiae University Hospital, D07 R2WY Dublin, Ireland; 2 Children’s Health Ireland, D01 X584 Dublin, Ireland; 3 Next Generation Sequencing Laboratory, Mater Misericordiae University Hospital, Eccles Street, D07 R2WY Dublin, Ireland; 4 Smurfit Institute of Genetics, Trinity College Dublin, University of Dublin, D02 VF25 Dublin, Ireland; 5 National Centre for Inherited Metabolic Disorders, Mater Misericordiae University Hospital, D07 R2WY Dublin, Ireland; 6 School of Medicine, Trinity College Dublin, University of Dublin, D02 P3X2 Dublin, Ireland; 7 School of Medicine, University College Dublin, D02 P3X2 Dublin, Ireland; 8 School of Physics, Clinical and Optometric Sciences, Technological University Dublin, D07 ADY7 Dublin, Ireland

**Keywords:** inherited retinal degenerations, retinal detachment, cataract, myopia, cystoid macular lesions

## Abstract

Background/Objective: Inherited retinal degenerations (IRDs) are the leading cause of blind registration in children and adults, yet 30–40% of cases remain genetically unresolved. Deep ophthalmic phenotyping may help to address this shortfall by identifying characteristic phenotypes. We describe the ophthalmic features of patients with stationary or progressive inherited retinal diseases other than outer retinal degeneration (i.e., secondary ophthalmic features, SOFs). Methods: This is a retrospective review of all patients attending an ophthalmic genetics clinic with a genetically confirmed IRD focusing on SOFs including refractive error, cataract, retinal detachment (RRD), cystoid macular lesions (CML) and epiretinal membrane (ERM). These features were assessed in the context of phenotype and genotype. Results: In a cohort of 429 genotyped patients, ≥1 SOFs were seen in 70.2% of patients, with 36.6% being affected by multiple SOFs. Refractive error (63.3%) and cataract (43.4%) were the most common secondary features, with a subset affected by CML (14.7%), ERM (10%) and RRD (4.7%). Conclusions: SOFs are common in patients with IRDs and most are amenable to therapeutic intervention even when no primary treatment (e.g., gene therapy) is available. We highlight patterns associated with genotypes and disease groups which may aid harmonisation of clinical and genetic diagnoses.

## 1. Introduction

Inherited retinal degenerations (IRDs) are a heterogeneous group of predominantly Mendelian genetic disorders which cause visual loss due to progressive outer retinal atrophy (e.g., retinitis pigmentosa, RP) or stationary dysfunction of synaptic activity (e.g., congenital stationary night blindness, CSNB). IRDs are the leading cause of blindness in children and working age adults in the Western world but, despite significant investment of resources, disease-modifying treatments are unavailable for most conditions [1,2].

Many IRDs have a final common pathway of outer retinal degeneration which may make correlation with a particular genotype difficult considering the >300 known genetic associations [3]. The extraocular features in syndromic IRDs may help to narrow the possible genetic aetiologies (e.g., hearing loss in Usher syndrome is associated with only 14 genes). Similarly, both syndromic and non-syndromic IRDs often have ophthalmic features other than those affecting the outer retina (i.e., photoreceptors and retinal pigment epithelium, RPE), which we refer to here as ‘secondary ophthalmic features’ (SOFs) [4]. These features (or constellations thereof) can be characteristic of phenotype groups (e.g., LCA, CSNB) or individual genotypes. Considering that 30–40% of IRDs currently remain unresolved, SOFs may significantly assist in discovery of new genetic associations and validation or exclusion of candidate variants in IRD-associated genes detected with next generation sequencing (i.e., pathogenic supporting, PP4, criteria of the American College of Medical Genetics and Genomics, ACMG) [5]. For example, cystoid macular lesions (CML) may occur in many IRDs [e.g., RP, X-linked (XL) retinoschisis (XLRS)], but if the patient also has high hyperopia, nanophthalmos and optic disc drusen, this significantly narrows the genotypic spectrum (e.g., *MFRP*, *BEST1*) [6].

The holy grail of IRD treatment is genetic rescue of retinal function, yet only a single disease-modifying gene therapy (voretigene neparvovec-rzyl, Luxturna for biallelic *RPE65*-IRD) has been approved though over 50 clinical trials of novel therapies (gene-specific and gene-agnostic) are in progress [7,8,9,10,11,12,13,14]. As opportunities to treat IRDs increase, further defining natural history and increasing the proportion of genetically resolved cases is relevant and SOFs may play a role in this.

Further to helping refine the phenotype, many SOFs (e.g., refractive error, cataract, CML, amblyopia) are amenable to simple and low-cost treatments which may stabilise or even improve visual function. Such interventions can often be made from the first clinical visit before a genetic diagnosis has been reached. While successful gene therapy trial outcomes are eagerly awaited, such simple interventions, when placed alongside appropriate supports (e.g., mobility, low vision, educational, financial, employment), may cause meaningful differences in activities of daily living and quality of life [15].

‘Target 5000’ is the Irish national registry of patients affected by IRDs which fulfils clinical (phenotyping, genetic testing, clinical management) and research (gene/variant discovery, therapy development) functions [15,16,17,18,19,20]. The goals of this paper are (1) to describe the SOFs of a large patient cohort with genetically confirmed IRDs, and (2) to identify any associations of SOFs with genotype or retinal phenotype and to discuss possible causes for these associations.

## 2. Materials and Methods

A retrospective cross-sectional review of patients with genetically confirmed IRDs was conducted with a focus on SOFs. This study was approved by the institutional review board of the Mater Misericordiae University Hospital (#1.378.1358) and follows the declaration of Helsinki. All patients provided informed consent.

The study considered patients recruited to the institutional IRD registry between July 2012 and July 2020. Patients were excluded if no genetic diagnosis was confirmed or if inadequate data were available. Of 540 patients with a clinical IRD diagnosis, genotype was confirmed for 429 (79.4%).

Assessed data included patient age, sex, clinical retinal diagnosis, genotype, best corrected visual acuity (BCVA), lens status (cataract or pseudophakia), refractive error (spherical equivalent and astigmatism, in dioptres, D), keratoconus (KC) diagnosis, presence of macular pathology [e.g., CML, epiretinal membrane (ERM)], retinal detachment (rhegmatogenous, Coats-like exudative vitreoretinopathy), amblyopia and glaucoma.

Patients were excluded from refractive error analysis if they had prior refractive surgery or were pseudophakic unless pre-operative refraction was available. BCVA was converted to LogMAR equivalent to facilitate analysis and FrACT equivalents were used for ‘counts fingers’ (1.9 LogMAR), ‘hand movements’ (2.3 LogMAR) and ‘perception of light’ (2.7 LogMAR) [21]. No light perception (NPL) vision was not quantified. Amblyopia was physician-reported in children (who represented the majority of cases with amblyopia) and self-reported in adults and validated by clinical findings. Refraction is specified as the spherical equivalent (SE, i.e., sphere power + 0.5 × cylinder power). Astigmatism was analysed independently based on cylinder power.

Phenotype groups were used for analysis, namely achromatopsia [ACHM, (*CNGA3*, *CNGB3*, *PDE6H*)], Bardet–Biedl syndrome [BBS (*BBS1*, *BBS4*, *BBS10*, *SDCCAG8*, *TRIM32*)], collagenopathies (*COL2A1*, *COL11A1*, *COLA18A1*), congenital stationary night blindness [CSNB (*CACNA1F*, *NYX*, *RDH5*, *TRPM1*)], Leber congenital amaurosis [LCA (*AIPL1*, *CEP290*, *CRB1*, *CRX*, *GUCY2D*, *PROM1*, *RDH12*, *RPE65*, *TULP1*)], non-syndromic RP [nsRP (*EYS*, *PRPF31*, *RHO*, *RP1*, *RP2*, *RPGR*, *SNRNP200*)] and Usher syndrome [USH (*ADGRV1*, *ARSG*, *CDH23*, *CLRN1*, *MYO7A*, *USH1C*, *USH2A*)]. All patients in this cohort with *BEST1*-associated retinopathy had autosomal dominant (AD) disease.

Statistical analyses were performed using SPSS v20 (IBM, Armonk, NY, USA). As there was similarity between eyes for BCVA (*p* = 0.218), only right eye values were analysed. Descriptive statistics (mean ± standard deviation, SD) were used to present quantitative data for all genotype and phenotype groups. SOFs were compared between phenotype groups and the most prevalent genotypes. For normally distributed data, comparisons of means were used and for non-normally distributed data, non-parametric tests were used. The limit of significance was set at *p* < 0.05.

## 3. Results

### 3.1. Demographics, BCVA and Amblyopia

The mean age of the 429 genetically characterised patients (from 317 pedigrees) was 39.8 ± 19.3 years, 44.7% were female and 81 different genotypes were represented (Table S1). Eight (1.9%) were carriers of XL IRD (*CHM*, *RPGR*), and the mean age of carriers was 42.9 ± 18.5 years. There were no significant age differences between sexes (*p* = 0.459).

**Figure 1:**
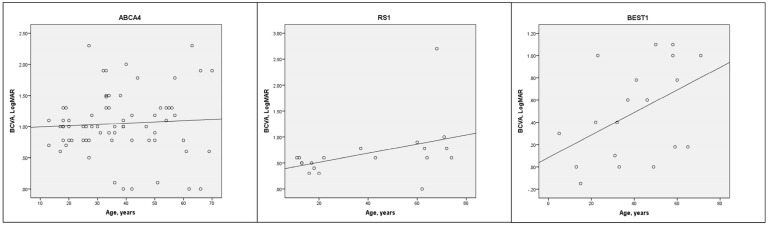
Cross-sectional scatter plots of BCVA vs. age (with trend line) for the three most prevalent genotypes (*ABCA4*, *RS1*, *BEST1*). Pearson correlation: *ABCA4* (r = 0.61, *p* = 0.617), *RS1* (r = 0.447, *p* = 0.55), *BEST1* (r = 0.425, *p* = 0.07).

Mean BCVA for the total cohort was 0.79 ± 0.73 LogMAR (Table 1 and Table S1). There were no significant BCVA differences between sexes (*p* = 0.236) but there was a correlation between worse BCVA and older age (r = 0.205, *p* < 0.001, Figure 1). The best BCVA was seen in the collagenopathy (0.20 ± 0.28 LogMAR) and CSNB (0.39 ± 0.37 LogMAR) groups while the worst BCVA was seen in the LCA (1.37 ± 0.85 LogMAR) and BBS (1.11 ± 0.91 LogMAR) groups. There was a significant BCVA difference between nsRP and BBS (*p* = 0.007) but not between nsRP and USH (*p* = 0.703). BCVA was similar between males and females for the *CHM* (*p* = 0.111) and *RPGR* (*p* = 0.340) genotypes. The blindness criteria for BCVA (≥1.0 LogMAR) were met by 31.0% and the driving standard (≤0.3 LogMAR) was met by 32.9%. Only four patients (0.9%, *CEP290*, *COL2A1* ×2, *RDH12*) had NPL vision, suggesting that IRDs rarely extinguish vision altogether.

Amblyopia was diagnosed in 88/429 (20.5%) and disproportionately affected females, 53/191 (27.7%), compared with males, 35/238 (14.7%), though this association was not significant (X^2^, *p* = 0.522). The IRD genotypes represented in the amblyopia group were those associated with retinitis pigmentosa (n = 30, 34.1%, *MYO7A*, *PRPF31*, *RHO*, *RPGR*, *SNRNP200*, *USH2A*), high myopia (n = 21, 23.9%, *COL2A1*, *RPGR*), LCA (n = 16, 18.2%, *CRB1*, *GUCY2D*, *RDH12*, *RPE65*) or significant hyperopia (n = 14, 15.9%, *BEST1*, *RS1*).

### 3.2. Cataract

Cataract and pseudophakia were diagnosed in 116 (27%) and 70 (16.3%) patients, respectively, together representing 43.4% of the cohort. There were no significant sex biases for cataract (*p* = 0.148) or pseudophakia (*p* = 0.977). Posterior subcapsular (n = 61, 52.6%), nuclear sclerotic (n = 24, 20.7%) and cortical (n = 17, 14.7%) cataract were similarly prevalent in either sex.

There were significant age differences between the cataract (47.5 ± 16.8 years) and pseudophakia (55.3 ± 14.4 years) groups when compared to the group without either (‘clear lens group,’ 30.3 ± 16.6 years, both *p* < 0.001). There was a significant BCVA difference between the clear lens (0.63 ± 0.58 LogMAR) and cataract (0.90 ± 0.86 LogMAR) groups (*p* = 0.017), but not between the clear lens and pseudophakia (0.86 ± 0.81 LogMAR, *p* = 0.071) groups. The combined group (i.e., cataract and pseudophakia together) was older (50.4 ± 16.4, *p* < 0.001) and had worse BCVA (0.89 ± 0.84, *p* = 0.003) than the clear lens group. There were also significant age (*p* < 0.001) and BCVA (*p* = 0.023) differences between the cataract and pseudophakic groups. One third of all cataracts in the cohort were identified in those ≤50 years, and 59% of the 41–50-year age group had cataract.

The USH and collagenopathy groups had the greatest rate of cataract (47.7%) and pseudophakia (40%), respectively, while ACHM had the lowest cataract (6.7%) and pseudophakia (0%) rate. No CSNB patient (*CACNA1F*, *NYX*, *RDH5*, *TRPM1*, mean age 26.7 ± 21.3 years) had cataract and only one (10%, *RDH5*, age 83 years) had pseudophakia. The relationship between lens status and genotype is summarized in Table 2 and Table S2.

### 3.3. Refractive Error

Refraction data were available for 226 patients (52.7%, 67 genotypes, Table S2). The refraction group was significantly younger (mean age 37.6 ± 19.5 years) than both the cataract (47.5 ± 16.8 years, *p* < 0.001) and pseudophakia (55.3 ± 14.4 years, *p* < 0.001) groups. Myopia (SE ≤ −0.50D) was present in 114 (50.4%) and hyperopia (SE > +0.75D) was present in 86 (38.1%). Significant refractive error (SE < −2.0 D or > +2.0 D) was present in 63.3% (n = 57 > +2.00D and n = 86 <−2.00D). There were no statistically significant sex differences for SE (*p* = 0.980), astigmatism (*p* = 0.451), high myopia (*p* = 0.739) or high hyperopia (*p* = 0.463). Age was not correlated with SE (*p* = 0.508), but was with astigmatism (r = −0.142, *p* = 0.033). SE and astigmatism were closely related, with a greater degree of hyperopia being associated with less astigmatism (r = −0.205, *p* = 0.002). BCVA did not correlate with SE (*p* = 0.200) or astigmatism (*p* = 0.778).

**Figure 2:**
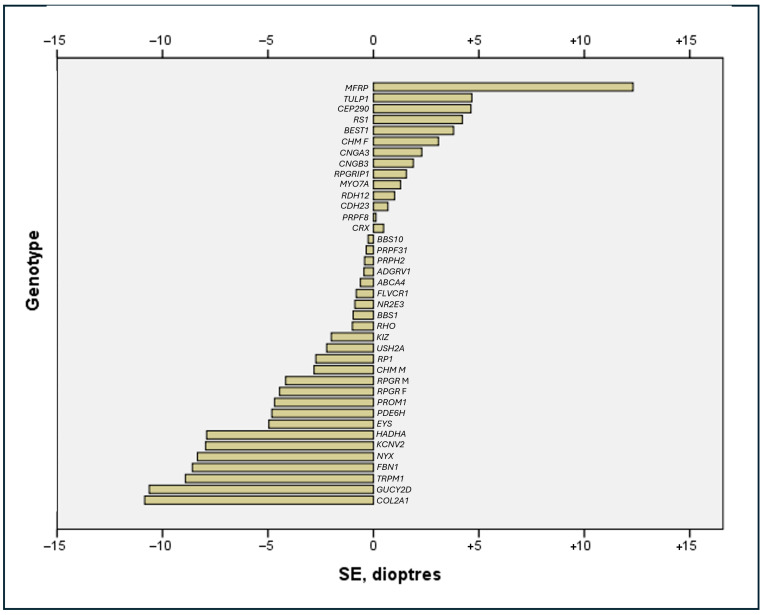
Bar chart of maximal refractive error stratified by genotype. SE = spherical equivalent refraction.

High myopia (≤−6.00D) was twice as common (n = 35, 15.5%) as high hyperopia (≥+5.00D, n = 17, 7.5%). Mean SE refraction was −9.54 ± 3.54D for high myopes and +7.36 ± 2.42D for high hyperopes, with the degree of ametropia (i.e., absolute deviation from emmetropia) being significantly greater for high myopes (*p* = 0.027). The clinically relevant differences in mean BCVA between high myopes (0.73 ± 0.74 LogMAR) and high hyperopes (1.21 ± 0.89 LogMAR) were not statistically significant (*p* = 0.053). There were no significant correlations between BCVA and the degree of high hyperopia (*p* = 0.633) or high myopia (*p* = 0.140). High myopia was associated with the *NYX* (n = 4, −8.34 ± 1.75D), *RPGR* (n = 3, −8.63 ± 2.69D) and *TRPM1* (n = 3, −8.92 ± 2.63D) genes. High hyperopia had the greatest representation from the *BEST1* (n = 4, +6.69 ± 0.97D), *RS1* (n = 4, +7.00 ± 2.00D), *MFRP* (n = 2, +12.31 ± 4.33D) and *TULP1* (n = 2, +6.13 ± 1.24D) genotypes. The degree of ametropia associated with each genotype is described in Figure 2, Table 2 and Table S2.

Among phenotype groups, there was a wide range of refractive error from extreme myopia to extreme hyperopia (Table 3). *COL2A1*-associated Stickler syndrome and the CSNB group had the most consistent refractive errors, with no patient being hyperopic.

Considering female ‘carriers’ of XL conditions and males with classic disease, there were no significant differences in SE or astigmatism for *RPGR* (*p* = 0.843 and *p* = 0.839, respectively) or *CHM* (*p* = 0.229 and *p* = 0.390, respectively). Despite their variable retinal phenotype, *RPGR* ‘carrier’ females were, on average, as myopic as their fully penetrant male counterparts.

Keratoconus was rarely reported (n = 5/429, 1.2%, *CEP290*, *GUCY2D*, *RHO*) in the total cohort but was disproportionately (n = 3/53, 5.7%) co-diagnosed with LCA (*CEP290* ×1, *GUCY2D* ×2). A history of corneal refractive surgery was documented in only two adults.

### 3.4. Macular Pathology

CML include both cystoid macular oedema and retinoschisis, which may be clinically difficult to distinguish despite their very different aetiologies. CML were present in 63 patients (14.7%). This was most prevalent in *RS1* (n = 15/21, 71.4%) and AD-RP [(n = 15/49, 30.6%), particularly *RHO* (6/16, 37.5%)] but less frequent in ARRP (3/14, 21.4%) and XLRP (1/24, 4.2%). ERM was present in 43 patients (10%), and most commonly encountered in those with RP (76.7%, n = 33/43) [BBS (n = 10/21, 47.6%), USH (n = 12/44, 27.3%) and non-syndromic RP (n = 11/86, 12.8%)]. There were no sex biases for CML (X^2^, *p* = 0.078) or ERM (*p* = 0.313). BCVA was significantly better in patients with CML (0.41 ± 0.37 vs. 0.84 ± 0.74, *p* < 0.001), though age was similar (40.0 ± 19.7 vs. 40.5 ± 18.6 years, *p* = 0.997). There were no differences for age (*p* = 0.865) or BCVA (*p* = 0.558) for those with and without ERM. Only one patient (0.2%, female) was recorded as having choroidal neovascularisation (CNV) with an AD *BEST1* phenotype (5% of *BEST1* group).

### 3.5. Retinal Detachment

Twenty-one patients (4.9%) had rhegmatogenous retinal detachment (RRD), with six having bilateral RRD. The most prevalent genotypes affected by RRD were *COL2A1* (n = 7, 33.3%), *FBN1* (n = 6, 28.6%) and *RS1* (n = 3, 14.3%). Only three patients (14.3%) with photoreceptor degenerations (*KCNV2*, *RDH12*, *USH2A*) had RRD (unilateral only). The proportions of each genotype affected by RRD were *COL2A1*: 53.8% (n = 7/13); *COL11A1*: 100% (n = 1/1); *COL18A1*: 100% (n = 1/1); *FBN1*: 50% (n = 6/12); *KCNV2*: 33.3% (n = 1/3); *RDH12*: 14.3% (n = 1/7); *RS1*: 14.3% (n = 3/21); and *USH2A*: 5.3% (n = 1/19). Bilateral RRD was only seen in collagenopathies/vitreoretinopathies with *COL2A1* (n = 2, 33.3%), *COL11A1* (n = 1, 16.7%), *COL18A1* (n = 1, 16.7%) and *FBN1* (n = 2, 33.3%) genotypes. The only hyperopic RRD patients (mean +3.79 ± 5.39D, range +2.75–+9.63D) had *RS1* genotypes (n = 3). Though only 25% of eyes with RRD had high myopia, the RRD group [−4.05 ± 8.01D (range −21.38–+9.63D)] was significantly more myopic than the non-RRD group (−0.87 ± 4.62D, *p* = 0.03). BCVA in eyes with treated RRD was not significantly worse than in those unaffected by RRD (0.98 ± 0.95 LogMAR vs. 0.79 ± 0.72, *p* = 0.813); however, two RRD eyes had NPL vision which was not quantified, thus underestimating the visual impact of RRD.

Twelve patients (2.8%) had documented retinal tears without RRD. These eyes had a mean refractive error of −4.50 ± 4.49D (range −0.25–−11.13D) and three of these patients (25%; *ABCA4*, *COL2A1*, *KCNV2*) had high myopia. Two female patients (0.5%; *CEP290*, *CERKL*) had a Coats-like exudative vitreoretinopathy (CLEVER) and one patient (0.2%, *RS1*) had retinal neovascularisation.

### 3.6. Glaucoma

Glaucoma was co-diagnosed in 18 patients (4.2%; *BEST1* ×2, *COL2A1* ×3, *FBN1* ×2, *FLVCR1*, *KIZ*, *MAK*, *MFRP*, *MYO7A*, *NR2E3*, *OAT*, *RHO* ×3, *RP1*, *USH2A*). Interestingly, (high) hyperopia (*MFRP*, *MYO7A*, *BEST1*) was more common in this group than myopia. Details of glaucoma medications were not available but only two patients (*BEST1*, *COL2A1*) required surgical management (tube shunt).

### 3.7. Cumulative SOFs per Patient

**Figure 3:**
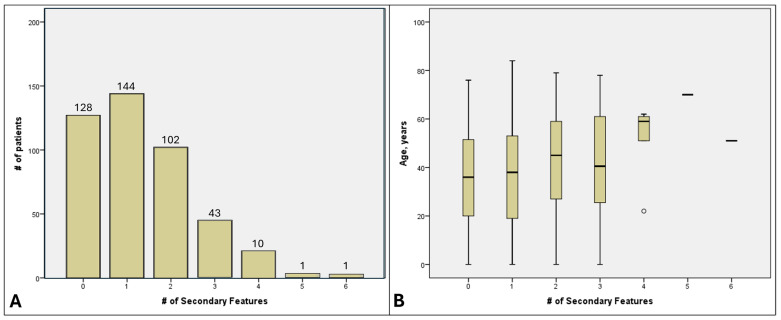
(**A**) Number of SOFs per patient. (**B**) Age boxplots of patients with different numbers of SOFs.

One or more SOFs (range 1–6) were seen in 301 (70.2%) patients, with 157 (36.6%) affected by ≥2 SOFs (Figure 3A). Even if refractive error was excluded, these proportions would be 62.9% (n = 270) and 31.0% (n = 133), respectively. Older patients had more SOFs, though this association was non-significant (*p* = 0.647, Figure 3B). Syndromic conditions with non-congenital vision loss, including the collagenopathy, USH and BBS groups, had the most SOFs while congenital conditions (ACHM, CSNB, LCA) had the fewest (Table S3). BCVA did not significantly vary between those with differing numbers of SOFs. Patients with NPL vision had mean 2.5 ± 1.7 SOFs.

## 4. Discussion

Secondary ophthalmic features are common in patients with IRDs, with 70% being affected by one or more SOFs. Refractive error (63.3%) and cataract (43.4%) were the most common secondary features, but a range of other vision-threatening secondary pathologies were also observed in this vulnerable group. Screening for and treatment of these SOFs may enable meaningful improvement in visual quality of life despite the lack of retinal disease-modifying therapies.

### 4.1. BCVA and Amblyopia

Considering the broad range of IRD genotypes included, BCVA varied widely. Expected BCVA differences between genotypes and phenotypes were corroborated [e.g., LCA has worse BCVA than RP; syndromic RP (BBS) has worse BCVA than nsRP] [22]. Female ‘carriers’ of pathogenic *RPGR* variants did not have significantly worse BCVA than males with classical disease, highlighting the fact that up to 1/3 of this group may have significant progressive visual disability [23].

There are multiple potential risk factors for amblyopia in IRDs including high ametropia/anisometropia, strabismus, early-onset cataract and asymmetrical retinopathy (e.g., female carriers of X-linked diseases). Amblyopia may be part of the working diagnosis while a child is being investigated for an IRD. Even after molecular confirmation, coexistent amblyopia may be a treatable feature. Confidence in an amblyopia diagnosis is difficult in adult IRD populations considering the coexistent structural abnormalities in the retina and that asymmetrical disease may simulate amblyopia, though the cases described herein were supported by clinical evidence [24].

### 4.2. Cataract

Cataract is a common SOF, diagnosed in 43.4% of the cohort studied here. A small central posterior subcapsular cataract may have a disproportionate impact on vision in the setting of visual field constriction (e.g., RP) or macular dysfunction (e.g., Stargardt disease) in which glare and defocus from cataract may compound image degradation [25]. The biological/metabolic dysfunction underlying lens pathology (i.e., cataracts or ectopia lentis) varies between retinal phenotypes. For instance, the posterior subcapsular cataract associated with RP may warrant surgery when gradual worsening causes reduced vision. In contrast, a patient with *FBN1*-associated Marfan syndrome may require lensectomy due to progressive lens subluxation despite having no ‘cataract’. Surgical treatment of cataract is commonly performed, seen in 16.3% of our total cohort. Cataract surgery in RP is often performed in the fifth to sixth decades, 20 years younger than in the general population [25,26]. In the current study, both the cataract and pseudophakia groups were significantly younger than the general population age at cataract surgery (range 60–80 years), highlighting the early onset of visually significant cataracts in IRDs. The optimal age for cataract surgery in IRDs is unknown but symptoms should guide case-by-case management. The one third of cataracts accounted for by the ≤50-year age group in the current cohort is 3× that of the general ≤50-year population (11%) [27].

Considering concomitant macular pathology, it is difficult to accurately predict the degree of BCVA improvement following cataract surgery though BCVA outcomes < 1.0 LogMAR are seen in 84% and a three-line (15-letter) improvement may be achieved in ~40% [25,28]. Certain intraoperative (e.g., zonular dialysis, 5%) and postoperative (e.g., posterior capsular opacification, 38%; worsened CML, 5%; capsular phimosis) complications are more likely in RP and should be actively anticipated and managed at the time of surgery [25,26,29].

Unlike the natural crystalline lens, most intraocular lens implants have no chromatic filtering capacity, and thus may allow transmission of more ultraviolet light, possibly accelerating oxidative stress and photoreceptor death. However, multiple reports describe improvement in function (BCVA and macular sensitivity) and no difference in disease progression compared with phakic counterparts [30,31,32,33,34]. Most data discuss cataract surgery in RP, where prognosis may be predicted by pre-operative preservation of the foveal ellipsoid zone (e.g., >600 µm width may predict a better outcome) and control of CML [32,33,35]. Tools such as patient-reported outcome measures may better clarify the additional qualitative benefits of cataract surgery in future studies [36,37].

### 4.3. Refractive Error

Significant refractive error is common in IRDs [38,39,40,41]. While the direct cause for this association has not been proven, degraded image quality due to photoreceptor degeneration is likely to disrupt normal emmetropization [42,43,44]. Unfortunately, these adaptive changes in refractive state may not improve BCVA due to progressive macular photoreceptor atrophy. In addition, considering the heterogeneous nature of this group of diseases, other individual or composite genetic factors may contribute to disruption of normal refractive development. Over 400 genes influence refractive error, some of which may be inherited as a haplotype (e.g., in East and South Asian ethnicities) or be influenced by behavioural trends (e.g., urban environments, increased near activities) [45]. The genetic influences on the development of myopia have previously been categorised by which biological process is interrupted [43]. In the case of IRDs, several of these categories are accounted for, including collagen fibril organisation/catabolism (e.g., *COL2A1*), retinal/lens development (e.g., *FBN1*, RP genes), secretory proteins [i.e., extracellular matrix (e.g., *IMPG1/IMPG2*) and cell–cell junctions (e.g., *RS1*)] and perception of visible light (e.g., *OPN1MW/OPN1LW*, *RHO*). Some genes implicated in IRDs (e.g., *BEST1*, *LRP5*, *MFRP*) affect organogenesis, modulating ocular development in the foetal and early childhood phases, leading to early-onset myopic or hyperopic refractive errors.

High myopia was significantly over-represented in this IRD population (15.5% prevalence) compared with the general population (5.3%), though this may vary with geography and ethnicity (NB the current population was predominantly white Irish) [46]. This suggests that, although myopia development is contingent on a legion of background genetic and environmental factors, pathogenic variants in some IRD-associated genes strongly influence myopic drive [41]. Less deleterious/hypomorphic alleles in these same genes may manifest as refractive error without retinal degeneration (e.g., female carriers of pathogenic *RPGR* variants) [43,47,48]. In fact, variants in IRD-associated genes are thought to underlie half of early-onset myopia cases [49]. Another marked example of IRDs and myopia is complete CSNB (~−6.8–−9.26D), which was replicated in the current cohort by *NYX* (−8.34 ± 1.75D) [40,41,50]. Other notable myopia associations include *RPGR* (~80% of XLRP, −2.6–−9D), *OAT* (gyrate atrophy, −9D), *RP1* (~1% of ARRP, −5.5D; 5–7% of ADRP, ~−1D) and *IMPG2* (~−4D) [51,52,53]. Our cohort largely concurred with these findings and highlights vitreoretinopathies (e.g., *COL2A1*, *FBN1*) as significant contributors to syndromic myopia parallel to photoreceptor and synaptic dystrophies. Hyperopia is common in some IRDs including AD *BEST1*-retinopathy, *RS1* and some LCA genotypes (in the current cohort: *AIPL1*, *ALMS1*, *CEP290*, *CRB1*, *CRX*, *TULP1*) [54,55]. Several genes (e.g., *BEST1*, *CRB1*, *MFRP*, *PRSS56* and *TMEM98*) which have roles in both foetal ocular development and post-natal outer retinal maintenance, can be associated with high hyperopia and short axial length [6,42,56,57].

Systematic assessment of refractive error not only enables correction of image defocus but may trigger investigations for IRDs (e.g., electroretinography, genetic testing) in children with high ametropia ±uncorrectable visual loss [39]. Additionally, a strong refractive phenotype paired with suggestive electrophysiological or fundus features may help to resolve candidate genetic variants (e.g., ACMG, PP4 criteria—highly suggestive phenotype for a single genetic aetiology) [5,20].

### 4.4. Keratoconus

Keratoconus (KC) can be considered a subtype of progressive refractive error, which may lead to corneal scarring, a further contributor to visual impairment. KC affects approximately 0.2% of the general population but its prevalence in IRDs is unknown, discussed only in infrequent case reports and an older (1968) genetically uncharacterised cohort study [58,59]. In the current cohort, KC was diagnosed in 1.2% (6× population risk). To our knowledge, this is the first study to estimate KC prevalence in a genetically characterised IRD population. Even if the implicated IRD gene has no direct corneal expression, developmental (e.g., mesenchymal differentiation) and behavioural (e.g., eye rubbing) factors may predispose to KC [60,61]. For example, in LCA the oculo-digital sign (i.e., eye rubbing to elicit visual sensations) may increase risk of corneal ectasia and its end stage sequelae (i.e., scarring) [60]. Another risk factor for KC which often overlaps with IRDs is intellectual disability, in which eye rubbing and/or atopy are common [62]. This refractive/media change further limits visual function and is a point of intervention, either in prevention by corneal collagen crosslinking or corneal transplantation where visual potential exists.

### 4.5. Macular Pathology

CML are well-described in RP and other IRDs (32% of paediatric and 59% of adult cohorts) [63,64,65,66]. In our study, the total CML rate was 14.7%—highest in XLRS (71.4%) and AD RP (30.6%). CML were less prevalent in severe early-onset disease (e.g., LCA, XLRP), in keeping with published reports [64]. The actual rate of CML may be higher as cystic spaces may resolve with treatment or collapse with disease progression and accurate visualisation/quantification may be limited (e.g., nystagmus, poor fixation, young children). The worse vision in those without CML is likely due to the higher prevalence of macular outer retinal atrophy in this group.

ERM is similarly common in RP (~20%) and the general population (10–28.9% of 45–84-year-olds), though RP patients with ERM were much younger (39.2 ± 18.1 years) [67,68]. Though RP has the best known association with ERM, 23.3% of the current cohort with ERM had non-RP genotypes. Whether this is directly related to the process of retinal degeneration or to usual ERM risk in the general population is unknown. ERM may be composed of fibroblasts, glial cells and/or RPE cells [69]. As the final stage of intraretinal pigment migration (i.e., bone spicules), RPE cells are thought to migrate transretinally (i.e., without retinal breaks) along the vasculature of the neurosensory retina to the inner retinal surface [70,71,72]. Clinically, the vitreous in RP is inundated with copious pigmented cells which are histologically confirmed as RPE cells [73]. Considering ERM is often a sign of advanced retinal degeneration, surgical correction of retinal distortion by ERM may have limited benefit, as demonstrated by Ikeda et al. who reported structural benefit without significant BCVA gain [74]. Integrity of foveal photoreceptors may be a useful prognostic guide for symptom-based management decisions on a case-by-case basis [74,75]. Interestingly, Stargardt disease, particularly with restricted macular atrophy, is infrequently associated with ERM [70], consistent with the current cohort where only 2.9% (n = 2/69, mean age 45.5 ± 29.0 years) of those with an *ABCA4* genotype had ERM. Perhaps the pan-retinal nature of RP-associated photoreceptor/RPE death has a more profound impact on RPE cell migration and metaplasia.

### 4.6. Retinal Detachment

The rate of RRD in the general population without a known genetic predisposition is approximately 1:10,000 or 0.01%; thus, the rate in this IRD cohort (n = 20/429, 4.7%) is 470× higher [76]. If collagenopathies (*COL2A1*, *COL11A1*, *COL18A1*) and Marfan syndrome are removed, this figure (n = 3/401, 0.7%) remains 70× the population risk. Several RRD risk factors are common in IRDs, namely myopia and abnormal vitreous. In the general RRD population, myopia is seen in 50–80% (region- and ethnicity-dependent), with greater myopia/axial length being positively correlated with RRD risk [71]. For example, lifetime RRD risk increases 10× with −3.00 to −5.00D of myopia or ~2.2% absolute RRD risk with ≤−5.00D of myopia increasing to 36% with coexistent lattice degeneration [76,77,78].

High myopia (≥−6DSE) was present in 15.5% of the current cohort, 3× the rate of the general population (5.3%) [46]. This does not match the 70–470× increased risk of RRD; thus, there are clearly other risk factors at play. Pruet et al. described a low incidence of RRD (1.8%) in an RP registry and postulated that early vitreous detachment, absence of lattice degeneration and unusually strong RPE-NSR adhesion might account for this relatively low rate (still 180×) [73]. The ‘adhesive’ force keeping the retina in place is derived from RPE pump function and thus diseases affecting the RPE may theoretically be more likely to incite RRD. However, RRD is clearly not exclusively Mendelian though strong family tendencies do occur, and a complex balance of risk factors (i.e., genetic and environmental) is at play [79].

Certain phenotypes/genotypes predispose to RRD, including XLRS (10–12.8% RRD rate) and Stickler syndrome type 1 (up to 73% RRD rate) [80,81]. The phenotype of the RRD (e.g., non-traumatic juvenile RRD, giant retinal tear) or the non-detached fellow eye (e.g., radial lattice, membranous vitreous anomaly) may suggest an IRD/vitreoretinopathy and instigate appropriate investigation, patient education and prophylaxis [82]. Similarly, confirmation of a pathogenic variant(s) in certain IRD genes (e.g., *COL2A1*) in as-yet asymptomatic relatives should prompt patient education, careful clinical surveillance ± prophylaxis.

RRD is related to pathological mechanical changes in the vitreoretinal interface (e.g., posterior vitreous detachment, PVD; lattice degeneration, 30–46% of RRD); thus, genetic variants in collagen genes (e.g., *COL2A1*, *COL11A1*, *COL18A1*) are logically implicated in Stickler syndrome [76,79,83]. Other genes have been associated with high myopia (e.g., *MYP* loci, *RDH5*, *RPGR*), axial elongation, lattice degeneration (e.g., *COL4A4*) and premature PVD [79,84]. The increased rate of cataract and higher rate of cataract surgical complications in IRD populations may further contribute to the risk of RRD. However, in this cohort, only 45% of patients presenting with RRD were pseudophakic but the design of this study precludes assessment of temporal associations.

**Figure 4:**
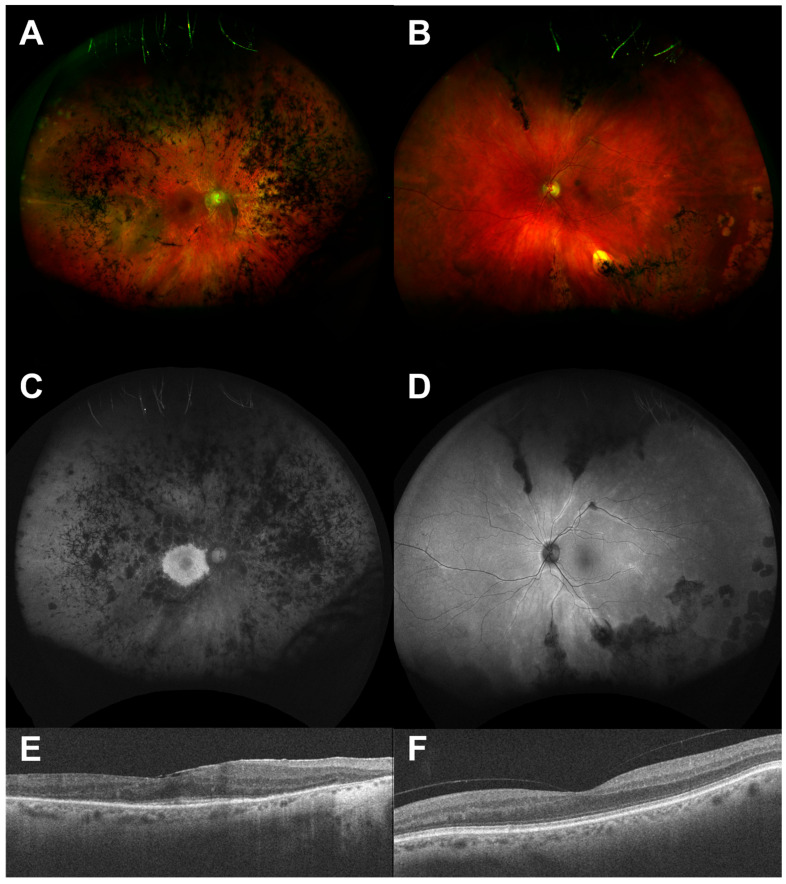
Asymmetrical pigmentary retinopathy after reattachment of RRD. The right eye (images (**A**,**C**,**E**), which had undergone successful retinal reattachment surgery, exhibits typical features of retinitis pigmentosa with sparing of only the central macula. The left eye (images (**B**,**D**,**F**)) shows much less marked retinal degeneration.

Though the occurrence of RRD in RP is low, primary surgical success and visual outcomes may be poorer than expected due to coexistent macular pathology [85]. Additionally, conventional treatment strategies (e.g., laser, cryotherapy) may be more (e.g., pigmentary retinopathy) or less (e.g., albinism) successful. Reattachment in an eye with underlying IRD may be accompanied by a dramatic pigmentary reaction and marked reduction in vision, out of keeping with the expected rate and degree of progression of retinal degeneration (Figure 4).

### 4.7. Glaucoma

Glaucoma was co-diagnosed in 4.2% of patients. This is not significantly different from the 3.5% population risk but may be an underestimation as symptoms (e.g., visual field constriction) and signs (e.g., optic atrophy) of IRDs may mask those of glaucoma [86]. The risk of this second vision-threatening pathology, particularly angle closure glaucoma, may be higher than that of the general population (1.1%) in some IRD genotypes (e.g., *BEST1*, *CRB1*, *MFRP*) associated with microphthalmia/nanophthalmos [56]. Surgical management of aggressive glaucoma may be more prone to complications (e.g., malignant glaucoma, progression), and novel therapeutic approaches may be required [87].

### 4.8. Rare SOFs

Several SOFs were noted extremely rarely in this population, namely CNV (0.2% vs. 7–50% in the literature), CLEVER (0.5% vs. 5% in the literature) and retinal neovascularisation (0.2% vs. 1–5% in the literature). However, diagnosis of these features may vary in different ethnicities or genotypes and detection depends on the investigations used (e.g., optical coherence tomography angiography is reportedly superior to fluorescein angiography for CNV detection) [88,89,90,91].

### 4.9. Limitations and Strengths

Limitations include the retrospective study design, low patient volumes for subgroup analyses and reliance on patient history and referral letters for ophthalmic and medical history. The creation of phenotype groups may be misleading as some genes are pleiotropic (i.e., associated with multiple phenotypes of varying severity). The study cohort was almost entirely of white Irish ethnicity; thus, the influence of the background genome on phenotype may differ in ancestrally distinct populations. This is a cross-sectional study including patients of varying ages and disease stages; thus, some secondary features may have not yet manifested (e.g., absence of cataract in early stage RP) or already resolved (e.g., CML resolution with topical carbonic anhydrase inhibitors), underestimating prevalence. CML may not have been present at the time of assessment, even in conditions known for CML (e.g., RP, *NR2E3*-retinopathy) as this cohort was not treatment-naïve. Disease duration is the ideal benchmark to measure SOF development, but this was not available in this dataset. The takeaway observation is that SOFs are common and often treatable and should be considered as a modifiable feature by clinicians.

Cataract surgery may be performed for reasons other than improving BCVA (e.g., angle closure, progressive ectopia lentis); thus, this may influence BCVA statistics for pseudophakic and phakic patients. Technical details of cataract surgery (e.g., intraoperative complications) were not available. BCVA was used as the main functional measure while visual field data (relevant in conditions affecting the retinal periphery) were not available; however, patient-reported outcome/experience measures may be more appropriate qualitative measures [30]. A strength of this study is that it reports on a large genetically resolved IRD population with a wide range of IRD genotypes and considers several clinically significant SOFs which may be amenable to treatment.

## 5. Conclusions

Secondary ophthalmic features of IRDs are common and may compound the visual impairment caused by primary retinal dysfunction, and multiple SOFs may overlap in a given individual. Although disease-modifying treatments for many of the common hereditary retinopathies are on the horizon, intervention for secondary features can start now to maximise visual function and quality of life. Patterns of these SOFs may enhance deep ophthalmic phenotyping to enable harmonisation with candidate genes and variants in line with existing advice (e.g., ACMG, ClinGen panels), aiding diagnosis.

## Figures and Tables

**Table 1:** Age, sex and BCVA of the 10 most prevalent genotypes.

**Ranking**	**Genotype**	***n* = (% of Total)**	**Age, Years ± SD**	**Female, *n* = (%)**	**BCVA, LogMAR Mean ± SD**
	Total	429 (100)	39.8 ± 19.3	191 (44.5)	0.79 ± 0.73
1	*ABCA4*	69 (16.2)	37.4 ± 15.5	40 (58.0)	1.08 ± 0.49
2	*RS1*	21 (4.9)	37.9 ± 24.9	0	0.72 ± 0.53
3	*BEST1*	20 (4.7)	39.5 ± 19.1	5 (25)	0.52 ± 0.44
3	*RPGR* (*Total*)	20 (4.7)	43.2 ± 21.3	8 (40)	0.69 ± 0.84
3M	*RPGR* (*Males*)	12 (2.8)	38.3 ± 25.7	0	0.79 ± 0.88
3F	*RPGR* (*Females*)	8 (1.9)	50.9 ± 13.3	8 (100)	0.57 ± 0.89
4	*USH2A*	19 (4.4)	46.4 ± 19.3	5 (26.3)	0.35 ± 0.41
5	*RHO*	16 (3.7)	42.3 ± 16.7	10 (62.5)	0.37 ± 0.25
6	*COL2A1*	13 (3)	27.0 ± 14.8	6 (46.2)	0.41 ± 0.39
7	*FBN1*	12 (2.8)	50.6 ± 17.5	6 (50)	0.69 ± 0.99
7	*PRPH2*	12 (2.8)	54.0 ± 14.3	4 (33.3)	0.32 ± 0.23
7	*RP1*	12 (2.8)	57.2 ± 13.5	8 (66.7)	0.48 ± 0.82

BCVA = best corrected visual acuity. F = female. LogMAR = logarithm of the minimum angle of resolution. M = male. SD = standard deviation.

**Table 2:** Cataract, pseudophakia and refraction for the most myopic and hyperopic genotypes with ≥5 patients.

Refractive Group	Genotype	Cataract, n = (%)	IOL, n = (%)	Refraction of n =	SE, Mean ± SD (D)	Cyl, Mean ± SD (D)	≤−6.00D, n = (%)	≥+5.00D, n = (%)
Myopia	*COL2A1*	1 (7.7)	4 (30.8)	4	−10.84 ± 7.64	2.44 ± 3.25	3 (75)	0
	*GUCY2D*	3 (27.3)	1 (9.1)	3	−10.63 ± 6.51	1.08 ± 1.13	2 (66.7)	0
	*TRPM1*	0	0	3	−8.92 ± 2.63	2.00 ± 0.50	3 (100)	0
	*FBN1*	3 (25.0)	8 (66.7)	3	−8.58 ± 7.26	1.83 ± 2.02	2 (66.7)	0
	*NYX*	0	0	4	−8.34 ± 1.75	2.56 ± 0.66	4 (100)	0
Hyperopia	*TULP1*	3 (100)	0	3	+4.67 ± 2.67	1.33 ± 0.76	0	1
	*RS1*	5 (23.8)	4 (19)	10	+4.21 ± 2.83	1.03 ± 1.20	0	4
	*BEST1*	5 (25.0)	0	12	+3.80 ± 2.38	0.73 ± 0.72	0	4 (33.3)
	*CHM* (Female)	0	1 (20.0)	3	+3.08 ± 0.69	0.67 ± 1.55	0	0
	*CNGA3*	0	0	3	+2.29 ± 2.35	0.75 ± 0.66	0	1 (33.3)

Cyl = astigmatism. D = dioptres. IOL = pseudophakia. SD = standard deviation. SE = spherical equivalent.

**Table 3:** Demographics, BCVA, lens status and refractive state of phenotype groups.

Phenotype	Total n =	Age (Years), Mean ± SD, (Range)	Male, n = (%)	BCVA, Mean ± SD, (Range)	Cataract, n = (%)	IOL, n = (%)	Refraction n = (%)	SE, in D, Mean ± SD (Range)	Cyl, Mean ± SD (D)	≤−6.00D, n = (%)	≥+5.00D, n = (%)
ACHM	15	20.3 ± 20.0 (2–64)	6 (40)	0.98 ± 0.28 (0.60–1.78)	1 (6.7)	0	13 (86.7)	+0.95 ± 4.26 (−7.5–+6.5)	1.29 ± 1.14	2 (15.4)	2 (15.4)
BBS	21	31.5 ± 11.9 (16–54)	13 (61.9)	1.11 ± 0.91 (0.00–2.70)	7 (33.3)	3 (14.3)	11 (52.4)	−0.70 ± 2.86 (−4.75–+2.75)	2.36 ± 1.35	0	0
CSNB	10	26.7 ± 21.3 (11–83)	7 (70)	0.39 ± 0.37 (−0.08–1.30)	0	1 (10)	8 (80)	−7.52 ± 3.53 (−0.05–−11.5)	2.22 ± 0.65	7 (87.5)	0
LCA	53	34.0 ± 19.2 (1–76)	30 (56.6)	1.37 ± 0.85 (−0.08–2.70)	16 (30.2)	4 (7.5)	19 (35.8)	+1.20 ± 6.59 (−14.75–+10.00)	1.04 ± 1.06	2 (10.5)	6 (31.6)
nsRP	86	45.3 ± 19.4 (8–78)	40 (46.5)	0.58 ± 0.73 (−0.18–2.70)	34 (39.5)	21 (24.4)	53 (61.6)	−2.05 ± 4.63 (−14.88–+15.38)	1.43 ± 1.05	7 (13.2)	2 (3.8)
COL	15	28.5 ± 14.9 (10–62)	7 (46.7)	0.20 ± 0.28 (−0.15–NPL)	1 (6.7)	6 (40)	5 (33.3)	−10.84 ± 7.64 (−3.75–−21.38)	2.44 ± 3.25	5 (100)	0
USH	44	45.4 ± 18.3 (13–84)	30 (68.2)	0.49 ± 0.65 (−0.08–2.70)	21 (47.7)	10 (22.7)	25 (56.8)	−0.55 ± 3.17 (−8.75–+4.25)	1.16 ± 0.68	2 (8)	0

ACHM = achromatopsia. BBS = Bardet–Biedl syndrome. BCVA = best corrected visual acuity in LogMAR. COL = collagenopathies. CSNB = congenital stationary night blindness. Cyl = astigmatism. D = dioptres. IOL = pseudophakia. LCA = Leber congenital amaurosis. NPL = no perception of light. nsRP = non-syndromic retinitis pigmentosa. USH = Usher syndrome.

## Data Availability

The data presented in this study are available on request from the corresponding author due to protection of confidentiality.

## Supplementary Materials

The following supporting information can be downloaded at https://www.mdpi.com/article/10.3390/genes16121433/s1: Table S1: Genotype, demographics and BCVA of IRD cohort ranked by genotype prevalence. Table S2: Demographics, BCVA, lens status and refractive state of all genotype groups. Table S3: Secondary features for each per phenotype group.

## Abbreviations

The following abbreviations are used in this manuscript:

BBS	Bardet–Biedl Syndrome
BCVA	Best corrected visual acuity
CML	cystoid macular lesions
CNV	choroidal neovascularisation
CSNB	congenital stationary night blindness
ERM	epiretinal membrane
IRDs	inherited retinal degenerations
KC	keratoconus
LCA	Leber congenital amaurosis
nsRP	non-syndromic retinitis pigmentosa
RP	retinitis pigmentosa
RRD	rhegmatogenous retinal detachment
SOFs	secondary ophthalmic features
USH	Usher syndrome
XLRS	X-linked retinoschisis
