# Supplementary material for: Secondary Ophthalmic Features Represent Diagnostic Clues and Potential Points of Intervention for Inherited Retinal Diseases (Target 5000 Report 3)
Source: Genes (Basel). 2025 Dec 1;16(12):1433. doi: 10.3390/genes16121433 (PMC12733187; doi:10.3390/genes16121433)
Supplement: Supplementary file 1 [file genes-16-01433-s001.zip › Supp Table S1.pdf]

Supplementary Table S1. Genotype, demographics, BCVA of IRD cohort ranked by genotype prevalence.

Ranking (top 10)	Genotype	n= (% of total)	Age, years ±SD	Female, n= (%)	BCVA, LogMAR mean ±SD
-	Total	429 (100)	39.8 ±19.3	191 (44.5)	0.79 ±0.73
1	*ABCA4*	69 (16.2)	37.4 ±15.5	40 (58.0)	1.08 ±0.49
2	*RS1*	21 (4.9)	37.9 ±24.9	0	0.72 ±0.53
3	*BEST1*	20 (4.7)	39.5 ±19.1	5 (25)	0.52 ±0.44
3	*RPGR*	20 (4.7)	43.2 ±21.3	8 (40)	0.69 ±0.84
	RPGR Males	12 (2.8)	38.3 ±25.7	0	0.79 ±0.88
	RPGR Females	8 (1.9)	50.9 ±13.3	8 (100)	0.57 ±0.89
4	*USH2A*	19 (4.4)	46.4 ±19.3	5 (26.3)	0.35 ±0.41
5	*RHO*	16 (3.7)	42.3 ±16.7	10 (62.5)	0.37 ±0.25
6	*COL2A1*	13 (3)	27.0 ±14.8	6 (46.2)	0.41 ±0.39
7	*FBN1*	12 (2.8)	50.6 ±17.5	6 (50)	0.69 ±0.99
7	*PRPH2*	12 (2.8)	54.0 ±14.3	4 (33.3)	0.32 ±0.23
7	*RP1*	12 (2.8)	57.2 ±13.5	8 (66.7)	0.48 ±0.82
	BBS1	11 (2.6)	29.5 ±11.8	4 (36.4)	0.95 ±0.87
	GUCY2D	11 (2.6)	39.1 ±10.5	8 (72.7)	1.33 ±1.02
	CHM	10 (2.3)	54.6 ±18.1	5 (50)	1.35 ±1.25
	CHM Male	5 (1.2)	58.8 ±13.4	0	1.96 ±1.05
	CHM Female	5 (1.2)	50.4 ±22.7	5 (100)	0.58 ±1.15
	CNGB3	10 (2.3)	23.8 ±23.3	7 (70)	1.00 ±0.33
	MYO7A	10 (2.3)	39.1 ±18.6	6 (60)	0.85 ±0.73
	ADGRV1	9 (2.1)	48.7 ±15.7	2 (22.2)	0.21 ±0.24
	CRX	8 (1.9)	39.3 ±16.4	1 (12.5)	1.04 ±1.08
	RDH12	7 (1.6)	39.1 ±20.1	6 (85.7)	1.53 ±0.80
	RPE65	7 (1.6)	32.9 ±29.8	2 (28.6)	1.08 ±0.97
	BBS10	6 (1.4)	27.0 ±6.93	2 (33.3)	1.52 ±0.99
	PRPF31	6 (1.4)	29.7 ±11.4	4 (66.7)	0.24 ±0.20
	CRB1	5 (1.2)	40.2 ±20.1	2 (40)	1.45 ±0.58
	EYS	5 (1.2)	48.8 ±17.7	5 (100)	0.34 ±0.17
	AIPL1	4 (0.9)	15.4 ±19.7	0	1.78*
	CEP290	4 (0.9)	19.5 ±17.3	2 (50)	1.1 ±1.13
	FLVCR1	4 (0.9)	37.3 ±17.9	2 (50)	0.37 ±0.12
	NYX	4 (0.9)	15.0 ±3.9	0	0.27 ±0.11
	PROM1	4 (0.9)	32.8 ±12.7	2 (50)	1.22 ±0.96
	SNRNP200	4 (0.9)	48.8 ±22.6	2 (50)	0.23 ±0.12
	CDH23	3 (0.7)	38.7 ±13.9	1 (33.3)	0.39 ±0.35
	CNGA3	3 (0.7)	10.5 ±4.9	1 (33.3)	0.95 ±0.07
	KCNV2	3 (0.7)	25.3±15.3	1 (33.3)	1.0 ±0.30
	Mitochondrial	3 (0.7)	56.0 ±13.0	3 (100)	0.20 ±0.28
	NR2E3	3 (0.7)	47.7 ±1.5	2 (66.7)	0.54 ±0.66
	OAT	3 (0.7)	54.3 ±8.1	1 (33.3)	0.87 ±1.24
	PRPF8	3 (0.7)	32.3 ±31.6	1 (33.3)	0.20 ±0.17
	TRPM1	3 (0.7)	23.3 ±6.4	2 (66.7)	0.39 ±0.21
	TULP1	3 (0.7)	34.0 ±4.6	1 (33.3)	2.30 ±0.57
	Rare genotypes	59 (13.8)	42.9 ±20.0	24 (40.7)	0.75 ±0.75

BCVA = best corrected visual acuity. LogMAR = logarithm of the minimum angle of resolution. SD = standard deviation. * = BCVA available for one patient only.

Rare genotypes were those represented by ≤2 patients: *ABCC6*, *ALMS1*, *ARSG, BBS4, C1QTNF5, C2ORF71, CACNA1F, CAPN5, CERKL, CFAP410, CFH, CLRN1, CNGB1, COL11A1, COL18A1, CTNNA1, DHX38, FRMD7, GUCA1A, HADHA, HK1, IFT140, KIZ, MAK, MERTK, MFRP, MTHFR, OPA1, PDE6H, PDE7B, PEX7, PRPF6, RDH5, RLBP1, RP1L1, RP2, RPGRIP1, SAG, SDCCAG8, TOPORS, TRIM32, USH1C, VHL.*
