# Supplementary material for: Secondary Ophthalmic Features Represent Diagnostic Clues and Potential Points of Intervention for Inherited Retinal Diseases (Target 5000 Report 3)
Source: Genes (Basel). 2025 Dec 1;16(12):1433. doi: 10.3390/genes16121433 (PMC12733187; doi:10.3390/genes16121433)
Supplement: Supplementary file 1 [file genes-16-01433-s001.zip › Supp Table S2.pdf]

Supplementary Table S2. Cataract, pseudophakia and mean refractive error for each genotype.

Genotype	Cataract, n= (%)	IOL, n= (%)	Refraction of n=	SE, mean ±SD (D)	Cyl, mean ±SD (D)	≤-6.00D	≥+5.00D
Total	116	70	226	-1.13 ±5.02	1.29 ±1.10	35	17
ABCA4	13 (18.8)	1 (1.4)	38	-0.61 ±2.60	0.93 ±0.91	2	0
ADGRV1	4 (44.4)	2 (22.2)	7	-0.45 ±1.48	0.90 ±0.77	0	0
AIPL1	0	1 (25)	1	+6.00	0	0	1
ALMS1	1 (50)	0	1	+7.50	-2.00	0	1
ARSG	1 (100)	0	1	+4.25	+0.50	0	0
BBS1	3 (27.3)	1 (9.1)	7	-0.96 ±2.89	2.07 ±1.46	0	0
BBS10	3 (50)	1 (16.7)	4	-0.25 ±3.19	2.88 ±1.11	0	0
BBS4	1 (50)	0	-	-	-	-	-
BEST1	5 (25.0)	0	12	+3.80 ±2.38	0.73 ±0.72		4
C1QTNF5	0	0	1	+0.25	0	0	0
C2ORF71	1 (100)	0	1	-14.88	0.75	1	0
CACNA1F	0	0	1	-0.05	1.50	0	0
CAPN5	0	1 (100)	1	-6	0	1	-
CDH23	0	1 (33.3)	2	+0.69 ±2.03	1.63 ±0.18	0	0
CEP290	1 (33.3)	0	2	+4.63 ±3.01	1.75 ±1.06	0	1
CERKL	0	0	1	+0.38	0.75	0	0
CFAP410	1 (50)	0	1	+1.25	1.50	0	0
CFH	0	1 (100)	-	-	-	-	-
CHM	3 (30)	1 (10)	7	-0.29 ±4.38	1.00 ±0.80	0	0
CHM Male	3 (60)	0	4	-2.81 ±4.26	1.25 ±1.04	0	0
CHM Female	0	1 (20)	3	+3.08 ±0.69	0.67 ±1.55	0	0
CLRN1	1 (100)	0	1	-8.00	1.50	1	0
CNGB3	0	0	8	+1.89 ±4.14	1.22 ±1.18	1	1
CNGA3	0	0	3	+2.29 ±2.35	0.75 ±0.66	0	1
CNGB1	0	0	1	0	0	0	0
CNNM4	1 (100)	0	1	+1.00	1.50	0	0

COL11A1	0	1 (100)	-	-	-	-	-
COL18A1	0	1 (100)	0	-	-	-	-
COL2A1	1 (7.7)	4 (30.8)	5	-10.84 ±7.64	2.44 ±3.25	5	0
CRB1	1 (20)	1 (20)	1	+10.00	0	0	1
CRX	0	1 (12.5)	3	+0.48 ±3.53	0.71 ±0.26	0	0
CTNNA1	0	0	-	-	-	-	-
DHX38	1 (100)	0	1	1.00	2.00	0	0
EYS	2 (40)	2 (40)	3	-4.96 ±3.76	1.00 ±0.90	1	0
FBN1	3 (25)	8 (66.7)	3	-8.58 ±7.26	1.83 ±2.02	2	0
FLVCR1	1 (25)	1 (25)	2	-0.81 ±0.97	2.63 ±0.88	0	0
FRMD7	0	0	-	'hyperopia'	-	-	-
GUCA1A	0	0	1	0.82	0.37	0	0
GUCY2D	3 (27.3)	1 (9.1)	3	-10.63 ±6.51	1.08 ±1.13	2	0
HADHA	0	0	2	-7.91 ±2.61	0.44 ±0.62	2	0
HK1	0	1 (100)	-	-	-	-	-
IFT140	0	0	-	-	-	-	-
KCNV2	1 (33.3)	0	3	-7.96 ±4.60	1.25 ±0.43	2	0
KIZ	1 (50)	0	2	-2.00 ±0.18	1.50 ±0.35	0	0
Mitochondrial	2 (66.7)	0	1	-6.00	1.50	1	0
MAK	1 (100)	0		-	-	-	-
MERTK	0	1 (100)	-	-	-	-	-
MFRP	1 (50)	1 (50)	2	+12.31 ±4.33	1.13 ±1.24	0	2
MTHFR	0	1 (100)	1	-6.375	1.75	1	0
MYO7A	4 (40)	3 (30)	7	+1.29 ±2.18	1.11 ±0.70	0	0
NR2E3	1 (33.3)	2 (66.7)	2	-0.88 ±2.83	1.50 ±1.06	0	0
NYX	0	0	4	-8.34 ±1.75	2.56 ±0.66	4	0
OAT	0	3 (100)	-	-	-	-	-
OPA1	0	0	1	-5.00	0	0	0
PDE6H	1 (50)	0	2	-4.81 ±2.74	2.38 ±1.24	1	0
PDE7B	0	1 (100)	-	-	-	-	-
PEX7	0	1 (100)	1	-3.00	0	0	0
PROM1	0	1 (25)	2	-4.69 ±4.51	1.63 ±0.88	1	0

PRPF6	2 (100)	0	1	+0.375	0.75	0	0
PRPF8	1 (33.3)	0	3	+0.50 ±1.08	1.17 ±1.01	0	0
PRPF31	3 (50)	1 (16.7)	4	-0.34 ±1.01	1.69 ±0.75	0	0
PRPH2	1 (8.3)	0	3	-0.42 ±0.95	1.33 ±0.58	0	0
RDH5	0	1 (50)	0	-	-	-	-
RDH12	4 (57.1)	0	3	+1.00 ±3.93	2.50 ±1.32	0	0
RHO	6 (37.5)	6 (37.5)	9	-1.00 ±3.99	1.11 ±0.69	1	0
RLBP1	0	0	1	-4.50	2.00	0	0
RP1	4 (33.3)	4 (33.3)	7	-2.73 ±2.89	0.69 ±0.40	1	0
RP1L1	1 (50)	0	1	-1.50	0.50	0	0
RP2	1 (50)	0	1	-15.38	2.25	1	0
RPE65	1 (14.3)	1 (14.3)	1	-2.00	1.00	0	0
RPGR Total	8 (40)	2 (10)	17	-4.28 ±2.78	2.32 ±1.11	3	0
RPGR Male	4 (33.3)	2 (16.7)	10	-4.16 ±3.19	2.38 ±1.18	2	0
RPGR Female	4 (50)	0	7	-4.45 ±2.28	2.25 ±1.09	1	0
RPGRIP1	2 (100)	0	2	+1.56 ±6.98	1.63 ±1.24	0	1
RS1	5 (23.8)	4 (19)	10	+4.21 ±2.83	1.03 ±1.20	0	4
SAG	0	1 (100)	-	-	-	-	-
SDCCAG8	0	1 (100)	-	-	-	-	-
SNRNP200	1 (25)	2 (50)	-	-	-	-	-
TOPORS	0	0	-	-	-	-	-
TRIM32	0	0	-	-	-	-	-
TRPM1	0	0	3	-8.92 ±2.63	2.00 ±0.50	3	0
TULP1	3 (100)	0	3	+4.67 ±2.67	1.33 ±0.76	0	1
USH1C	0	1 (100)	-	-	-	-	-
USH2A	10 (52.6)	4 (21.1)	8	-2.22 ±3.16	1.31 ±0.70	1	0
VHL	-	-	-	-	-	-	-

Cyl = astigmatism. D = dioptres. IOL = pseudophakia. SD = standard deviation. SE = spherical equivalent.

All refractions are of phakic eyes only.
