# Supplementary material for: Secondary Ophthalmic Features Represent Diagnostic Clues and Potential Points of Intervention for Inherited Retinal Diseases (Target 5000 Report 3)
Source: Genes (Basel). 2025 Dec 1;16(12):1433. doi: 10.3390/genes16121433 (PMC12733187; doi:10.3390/genes16121433)
Supplement: Supplementary file 1 [file genes-16-01433-s001.zip › Supp Table S3.pdf]

Supplementary Table S3. Secondary features for each phenotype group.

Phenotype	Total n=	Age (years), mean ±SD, (range)	BCVA, mean ±SD, (range)	Amblyopia	RE >2.00D	Cataract, n= (%)	IOL, n= (%)	CML	ERM	RRD	Glaucoma	KC	Mean SF
ACHM	15	20.3 ±20.0 (2 – 64)	0.98 ±0.28 (0.60 – 1.78)	1 (6.7)	4 (26.7)	1 (6.7)	0	0	0	0	0	0	0.83
BBS	21	31.5 ±11.9 (16 – 54)	1.11 ±0.91 (0.00 – 2.70)	1 (4.8)	8 (38.1)	7 (33.3)	3 (14.3)	2 (9.5)	12 (57.1)	0	0	0	1.6
CSNB	10	26.7 ±21.3 (11 – 83)	0.39 ±0.37 (-0.08 – 1.30)	1 (10)	8 (80)	0	1 (10)	0	1 (10)	0	0	0	1.1
LCA	53	34.0 ±19.2 (1 – 76)	1.37 ±0.85 (-0.08 – 2.70)	8 (15.1)	17 (32.1)	16 (30.2)	4 (7.5)	1 (1.9)	2 (3.8)	0	0	3 (5.7)	1
nsRP	86	45.3 ±19.4 (8 – 78)	0.58 ±0.73 (-0.18 – 2.70)	8 (9.3)	30 (34.9)	34 (39.5)	21 (24.4)	17 (19.8)	14 (16.3)	0	2 (2.3)	1 (1.2)	1.4
COL	15	28.5 ±14.9 (10 – 62)	0.20 ±0.28 (-0.15 – NPL)	4 (26.7)	6 (40)	1 (6.7)	6 (40)	1 (6.7)	1 (6.7)	8 (53.3)	3 (20)	0	2
USH	44	45.4 ±18.3 (13 – 84)	0.49 ±0.65 (-0.08 – 2.70)	1 (2.3)	15 (34.1)	21 (47.7)	10 (22.7)	11 (25)	13 (29.5)	0	2 (4.5)	0	1.7

ACHM = achromatopsia. BBS = Bardet-Biedl syndrome. BCVA = best corrected visual acuity in LogMAR. COL = collagenopathies. CSNB = congenital stationary night blindness. Cyl = astigmatism. D = dioptres. IOL = pseudophakia. LCA = Leber congenital amaurosis. NPL = no perception of light. nsRP = non-syndromic retinitis pigmentosa. RE = refractive error. SF = secondary feature. USH = Usher syndrome.
